# Chemical Composition, Antioxidant and Anticancer Activities of *Leptocarpha rivularis* DC Flower Extracts

**DOI:** 10.3390/molecules26010067

**Published:** 2020-12-25

**Authors:** Iván Montenegro, Jorge Moreira, Ingrid Ramírez, Fernando Dorta, Elizabeth Sánchez, Juan Felipe Alfaro, Manuel Valenzuela, Carlos Jara-Gutiérrez, Ociel Muñoz, Matias Alvear, Enrique Werner, Alejandro Madrid, Joan Villena, Michael Seeger

**Affiliations:** 1 Escuela de Obstetricia y Puericultura, Facultad de Medicina, Universidad de Valparaíso, Angamos 655, Reñaca, Viña del Mar 2520000, Chile; jorge.moreira@postgrado.uv.cl; 2 Centro de Biotecnología “Dr. Daniel Alkalay Lowitt”, Universidad Técnica Federico Santa María, Avda. España 1680, Valparaíso 2390123, Chile; ingrid.ramirez@usm.cl (I.R.); fernando.dorta@usm.cl (F.D.); elizabeth.sanchez@usm.cl (E.S.); felipealfaro88@gmail.com (J.F.A.); 3 Laboratorio de Microbiología Celular, Instituto de Investigación e Innovación en Salud, Facultad de Ciencias de la Salud, Universidad Central de Chile, Santiago 8320000, Chile; manuel.valenzuela@ucentral.cl; 4 Centro de Investigaciones Biomédicas (CIB), Laboratorio de Estrés Oxidativo, Escuela de Kinesiología, Facultad de Medicina, Universidad de Valparaíso, Viña del Mar 2520000, Chile; carlos.jara@uv.cl; 5 Institute of Food Science and Technology, University Austral of Chile, Valdivia 5090000, Chile; ocielmunoz@uach.cl; 6 Laboratory of Industrial Chemistry, Process Chemistry Centre, Åbo Akademi University, Biskopsgatan 8, FIN-20500 Turku/Åbo, Finland; matias.alvear@abo.fi; 7 Departamento de Ciencias Básicas, Campus Fernando May, Universidad del Bío-Bío, Avda. Andrés Bello 720, Casilla 447, Chillán 3780000, Chile; ewerner@ubiobio.cl; 8 Laboratorio de Productos Naturales y Síntesis Orgánica (LPNSO), Departamento de Química, Facultad de Ciencias Naturales y Exactas, Universidad de Playa Ancha, Avda. Leopoldo Carvallo 270, Playa Ancha, Valparaíso 2340000, Chile; 9 Centro de Investigaciones Biomédicas (CIB), Facultad de Medicina, Campus de la Salud, Universidad de Valparaíso, Angamos 655, Reñaca, Viña del Mar 2520000, Chile; 10 Laboratorio de Microbiología Molecular y Biotecnología Ambiental, Departamento de Química, Universidad Técnica Federico Santa María, Avda. España 1680, Valparaíso 2390123, Chile

**Keywords:** palo negro, antioxidant, cancer cell line

## Abstract

An evaluation of antioxidant and anticancer activity was screened in *Leptocarpha rivularis* DC flower extracts using four solvents (*n*-hexane (Hex), dichloromethane (DCM), ethyl acetate (AcOEt), and ethanol (EtOH)). Extracts were compared for total extract flavonoids and phenol contents, antioxidant activity (2,2-diphenyl-1-picryl-hydrazyl-hydrate (DPPH), ferric reducing antioxidant potential (FRAP), total reactive antioxidant properties (TRAP) and oxygen radical absorbance capacity (ORAC)) across a determined value of reduced/oxidized glutathione (GSH/GSSG), and cell viability (the sulforhodamine B (SRB) assay). The most active extracts were analyzed by chromatographic analysis (GC/MS) and tested for apoptotic pathways. Extracts from Hex, DCM and AcOEt reduced cell viability, caused changes in cell morphology, affected mitochondrial membrane permeability, and induced caspase activation in tumor cell lines HT-29, PC-3, and MCF-7. These effects were generally less pronounced in the HEK-293 cell line (nontumor cells), indicating clear selectivity towards tumor cell lines. We attribute likely extract activity to the presence of sesquiterpene lactones, in combination with other components like steroids and flavonoids.

## 1. Introduction

*Leptocarpha rivularis* DC (family, Compositae; tribe, Heliantheae; presently classified as Asteraceae), locally known as “palo negro”—or, in Mapudungun, Cüdu-mamëll, originally named by the Mapuche people of Chile—is a perennial shrub native to southern Chile which grows in humid and sunny soils up to two meters in height, with toothed-edged leaves up to 10 cm. Although it is a typical species of the “Valdivian forest”, distributed from the Region of Maule to the Region of Los Lagos, it also grows in the coastal and Andean mountain ranges [1].

**Figure 1:**
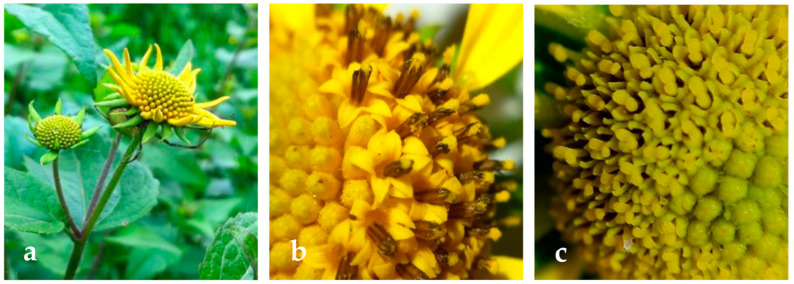
Flowers of *L*. *rivularis* plants grown in Araucania Region, South Chile (**a**–**c**).

This plant has hermaphroditic flowers up to 2 cm in diameter, composed of axillary heads and yellow terminals. The head is surrounded by two rows of herbaceous bracts that protect the flower in the button state [2]. Hoffman describes *L. rivularis* flowers (Figure 1a–c) as ligulate on the border, and tubular in the center [3]. In ligulate, or peripheral, flowers, the ovary is atrophied and the remaining parts of the flower are completely missing. In the central, or discoidal, flowers, there are two distinct groups: male flowers and female flowers.

Parts of this shrub have been widely used since pre-Hispanic times in traditional Mapuche medicine for gastrointestinal and stomach ailments and is now widely sold in local markets and in pharmacies for cancer prevention. The secondary metabolite characteristic of this family includes acetylenics, sesquiterpenes, and sesquiterpene lactones. *L. rivularis* also contains essentials oils, flavonoids, and triterpenes [4]. Despite the great interest in *Leptocarpha rivularis* DC due to the wide variety of secondary metabolites with biological activity that it produces—as in all plant extracts containing antineoplastic activity profusely studied in recent decades—descriptions of flower extracts, composition, antioxidant capacity, or cytotoxic effect against cancer cell lines are still incomplete. This paper, then, reports on the antioxidant capacity and cytotoxic effects of *Leptocarpha rivularis* DC flower extracts.

Although the properties of its flowers have yet to be exhaustively catalogued, previous partial phytochemical studies of this plant have shown the presence of sesquiterpenes. Of these, notable is the main active heliangolide (sesquiterpene lactone) compound “Leptocarpin”, an inhibitor of NF-kappa B (and therefore, cytotoxic to cancer cells) [5]. Mostly quantified in its leaves and reported as responsible for some of the plant’s antioxidant properties, essential oils of “palo negro” have also been shown to be a source of lipophilic compounds, namely terpene alpha-thujone, the sesquiterpenes beta-caryophyllene and caryophyllene oxide. UACH (Universidad Austral de Chile) researchers have also reported that the active ingredient present in *L. rivularis*, Leptocarpin, modulates the activity of MDR (multidrug resistance) proteins by functional interaction with MRP1 (multidrug resistance protein), resulting in an increase in the accumulation of rhodamine 123 (R123) in oligodendroglioma cells and an increase in sensitivity to cytotoxins [6]. Furthermore, hypoglycemic assays with an aqueous extract of *L. rivularis* showed a strong decrease in blood glucose levels in Sprague-Dawley rats [7]. Moreover, studies of combined *L. rivularis* flower and leaves determined a significant increase in the enzymatic level of the glutathione system [7], and sequential extracts obtained from *L. rivularis* flowers have been shown to have significant antioxidant capacity, with semipolars presenting the best antioxidant results in vitro. [8].

## 2. Results

### 2.1. Extract Yields

The sequential soxhlet extraction method was adapted to obtain different flower extracts using the following solvents: hexane (Hex), dichloromethane (DCM), ethyl acetate (AcOEt) and ethanol (EtOH). The highest yields were achieved with ethanol 15.52% (*w*/*w*), followed by hexane 6.50% (*w/w*), dichloromethane 5.80% (*w*/*w*), and ethyl acetate 4.60% (*w*/*w*).

### 2.2. Phytochemical Content

After flower extracts were obtained, the phytochemical content (i.e., total phenolic contents, flavonoids, anthraquinone) was measured using colorimetric assays as summarized in Table 1. Total anthraquinone and flavonoids showed significant differences in EtOH extracts (*p* < 0.05) over other extracts. Total phenols (TPC) of flower extracts are expressed in terms of GAE mg L^−1^ and presented in Table 1. The total phenolic content in both the Hex and EtOH extracts showed significant differences between them and with the other extracts evaluated (*p* < 0.05). TPCs were calculated using the following linear regression equation obtained from the standard plot of gallic acid: y = 0.001x + 0.021, r_2_ = 0.998.

#### 2.2.1. Total Antioxidant Activity

Antioxidant activity of *L. rivularis* flower extracts was evaluated in a series of in vitro DPPH, FRAP, TRAP, and ORAC assays (see Table 2). The DPPH assay showed that the Hex extract had poor activity (*p* < 0.05) compared to the positive control (Trolox (T). DCM was the most active extract compared to AcOEt and EtOH, but less active than T (*p* < 0.05). The FRAP assay showed that DCM, AcOEt and EtOH extracts had better antioxidant activity than positive controls (*p* < 0.05). Under TRAP, the Hex extract was the least active compared with positive controls (Gallic acid (GA) and Butylhydroxytoluene (BHT), *p* < 0.05).

The ORAC rate—which is widely used to evaluate concoction antioxidants, and measures the protection supplied by an antioxidant to an objective molecule oxidized by peroxyl radicals—showed significant differences between AcOEt (Table 2) and *n*-Hexane and ethanol extracts, the latter two of which presented lower activity. AcOEt, in contrast, presented major ORAC results. This, however, had little correlation with the phenolic compound contents (r_2_ < 0.5).

#### 2.2.2. Reduced Glutathione Assay

The ratio of reduced GSH to oxidized GSH (GSSG) is an indicator of cellular health, with reduced GSH constituting up to 98% of cellular GSH under normal conditions. Table 3 shows the results from the reduced glutathione assay using pooled flower extracts of *L. rivularis* under the different solvents analyzed. Ethanol extracts had significantly higher GSH content compared with the other solvents, however, the content of GSSG was also significantly higher (4 times over AcOEt, and 10 times over *n*-hexane and DCM). Thus the DCM extract had the best GSH/GSSG ratio content—higher than other extracts, though significantly—and the lowest GSH to GSSG conversion rate.

#### 2.2.3. GC-MS Analysis

The most active and selective *L. rivularis* flower extracts against breast and prostate cancer cell lines (*n*-Hexane, DCM, AcOEt) were analyzed by chromatographic analysis (GC/MS). The results of this analysis are shown in Table 4, Table 5 and Table 6. The *n*-Hexane extract contained high amounts of fatty acids and derivatives, including diterpene 4,8,13-Duvatriene-1,3-diol, ß-Amyrin acetate, and triterpene esters lupeol acetate (41.44% of the total extract composition, see Table 4). The DCM extract had diterpene thunbergol, and long chain alkanes tetratriacontane and 1-Heptatriacotanol, and the triterpenoids ß-Amyrin acetate, lupeol acetate, and (43.47% of the total composition, see Table 5). The AcOEt extract had terpene compounds, including Leptocarpin, 4,8,13-duvatriene-1,3-diol, the sterol stigmasterol, fenretinide (amino phenol diterpene derivative), and linear alcohol 1-Heptatriacotanol (55.93% of the total extract composition, see Table 6.

### 2.3. Cytotoxic Activity

The Hex, DCM and AcOEt flower extracts were determined to be rich in sesquiterpenes, sesquiterpene lactones and other natural products (caryophyllene oxide, dehydrocostus lactone, etc., Table 4, Table 5 and Table 6). Cytotoxicity of these flower extracts was evaluated in vitro against different cancer cell lines: HT-29 (colon cancer); PC-3 (prostate cancer); MCF-7 (breast cancer); and HEK-293 (a nontumor human embryonic kidney cell line). A sulforhodamine B colorimetric assay was set up to obtain the IC_50_ values of the tested extracts, using eight concentrations (from 0.625 to 62.5 µg/mL; save ethanol extract, at concentrations from 0.625 to 250 µg/mL) for each extract, in triplicate. Results are shown in Table 7. Interestingly, according to our data, at nontoxic concentrations against nontumor cells, all the extracts studied reduced the cellular viability of cancer cells. Cancer cell line IC_50_ values are in the range of 3.0—8.8 µg/mL, with the exception of ethanol extract, above 91 µg/mL. The lowest IC_50_ value (highest cytotoxicity) was against HT-29. Flower extracts were much less cytotoxic against HEK-293 (nontumor), with IC_50_ values above 82.9 µg/mL.

### 2.4. Selective Index (SI)

Table 8 shows selectivity results for flower extracts, which (with the exception of ethanol extract) were shown to act selectively against cancer cells. Interestingly, the inhibitory effect of flower extracts on cancer cell viability is at the same order of magnitude as leptocarpin—the sesquiterpene lactone isolated from *L. rivularis*—but with a selectivity one order of magnitude higher [5].

**Figure 2:**
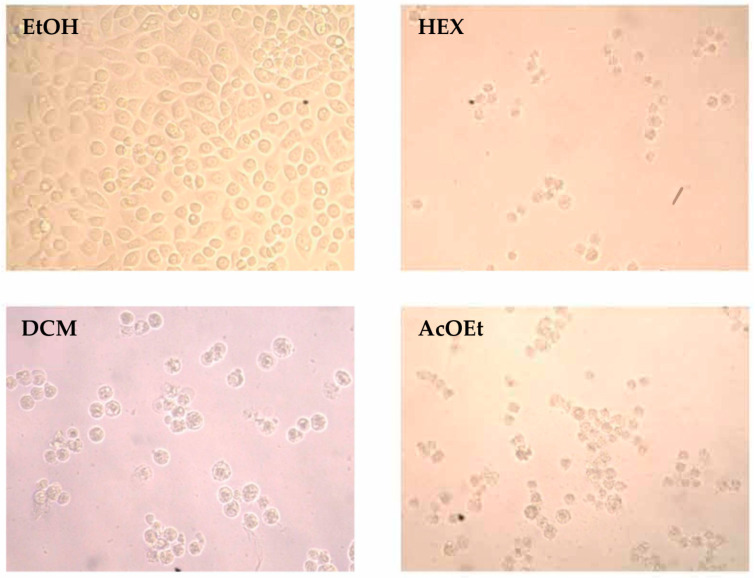
Effect of extracts on cellular morphology in HT-29 cells. Representative photographs presented here show cellular morphologic changes observed by contrast phase microscopy of cells under: EtOH (solvent control); Hex, hexane extract; DCM, dichloromethane extract; AcOEt, ethyl acetate extract.

Given their inhibitory effects against cancer cell viability and high selectivity index, hexane, dichloromethane, and ethyl acetate extracts were analyzed in greater detail on the cell lines tested. To elucidate the mechanism by which flower extracts reduced cell viability in the cell lines tested (HT-29, MCF-7 and PC-3 cells), cell morphology changes were observed under contrast phase microscope (200×). Figure 2 shows representative photographs of HT-29 colon cancer cells.

Exposure to Hex, DCM and AcOEt extracts (37.5 μg/mL for 48 h) significantly affected cellular morphology and total number of cells versus ethanol-treated cells (control), with a rounded morphology suggestive of a loss of cellular adhesion and a cell death process. These results agree with previous studies (Table 7) showing that cytotoxicity increased against studied cancer cell lines after flower extract treatments.

Mitochondrial membrane permeability has been suggested as playing an important role in activating cellular apoptosis [10]. To check the effect of Hex, DCM and AcOEt extracts on mitochondrial membrane permeability, rhodamine 123 staining was carried out in treated HT-29, MCF-7, and PC-3 cancer cells and nontumor HEK-293 (6.25 and 37.5 μg/mL). The percentages of rhodamine 123-stained cells are summarized in Table 9.

**Figure 3:**
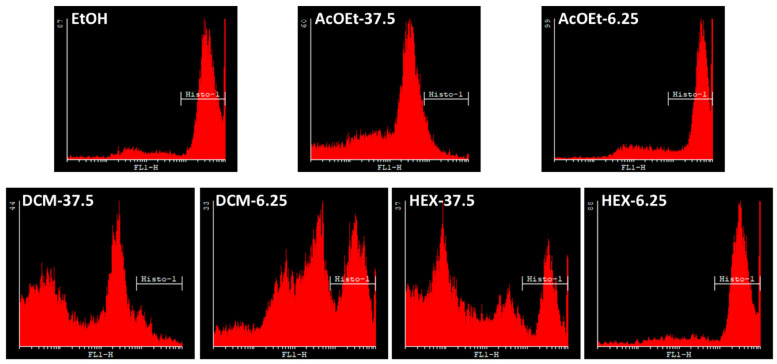
Histogram showing mitochondrial membrane permeability after treatment with different concentrations of the flower extracts in HT-29 cells. Extract-treated (6.25 and 37.5 μg/mL) HT-29 cells were stained with rhodamine 123 and subjected to flow cytometry analysis. Hex, hexane; DCM, dichloromethane and AcOEt, ethly acetate; EtOH, ethanol solvent control cells.

As shown in Figure 3, all extracts decreased the mitochondrial membrane permeability, with the largest decrease in HT-29 cells treated with DCM extract. Furthermore, the mitochondrial function affected by Hex, DCM and AcOEt flower extracts is shown to be dependent on concentration. These results are in line with cell viability assays (Table 7) and contrast phase microscopy (Figure 2), and are supported by previous studies demonstrating leptocarpin-induced apoptosis in cancer cell lines through permeabilization of the mitochondrial membrane [5].

Finally, we propose that changes in mitochondrial membrane permeability produced by the extracts alter the mitochondrial functionality, dissipating the proton gradient or altering electronic transport and inducing apoptosis by activation of caspases.

**Figure 4:**
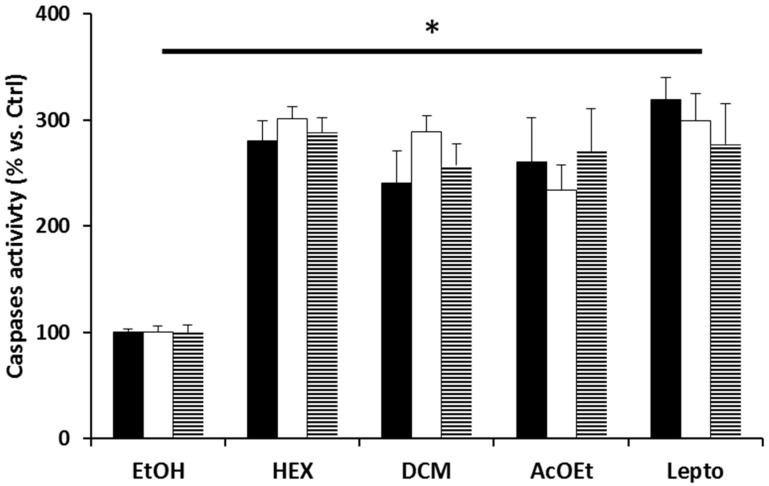
Effect of extracts on caspase activation of different cell lines. Activation of caspases in MCF-7 (black bars), PC-3 (white bars) and HT-29 (dashed bars) cells treated with Hex, DCM and AcOEt flower extracts (37.5 μg/mL). Data are reported as the ratio of activities of treated cells and ethanol-treated cells (control), which were arbitrarily assigned a unitary value. Leptocarpin (Lepto) is used as control positive. Data are reported as mean values ± SD of three different experiments with samples in triplicate, * *p* < 0.05; significantly different from the ethanol-treated cells.

Next, caspase activation was measured to assess its inhibitory effect on cells. Figure 4 shows caspase activation was higher in cells exposed to Hex, DCM and AcOEt flower extracts than in control, further showing cell death by apoptosis. Indeed, the increased caspase activity in all three extracts is an indication that flower extracts function by leptocarpin-induced caspase activation.

On the whole, all the three extracts showed an increase in caspase activity indicating the activation of the apoptotic pathway. These data show the Hex, DCM and AcOEt flower extracts, as leptocarpin, induced caspase activation.

Finally, we think that the changes in mitochondrial membrane permeability produced by the extracts alter the mitochondrial functionality, dissipating the proton gradient or altering electronic transport and inducing apoptosis by activation of caspases.

## 3. Discussion

The phenolics, anthraquinones and flavonoids present in *L. rivularis* flowers were evaluated. Phenolic compounds are important due to their multifunctional pharmacological properties. These classes act broadly as natural antioxidants due to their oxygen-quenching, redox, and metal-chelating abilities. Thus plant extracts rich in phenolic compounds are often associated with an abundant range of therapeutic and physiological benefits [11]. Anthraquinones are an important class of natural products found in various plant species, displaying remarkable bioactive properties, including anticancer, antitumor, anti-inflammatory, antiarthritic, antifungal, antibacterial, and antimalarial activities, among others [12]. Additionally, various kinds of flavonoids can promote apoptosis in cancer cells [13,14,15]; quercetin, a flavonol, has been reported as an anticancer substance against prostate and breast cancers [16,17]. Another study, with flavonoids Gliricidin7-O-hexoside and Quercetin 7-O-rutinoside from the bird’s-nest fern (*Asplenium nidus*), reports their chemopreventive potential against human hepatoma HepG2 and human carcinoma HeLa cells [17].

DPPH, FRAP, TRAP and ORAC assays indicated *L. rivularis* flower extracts as potentially rich sources of natural antioxidants. Although ethanolic extract was a good source of phyto-constituents, with a high total content of phenols, flavonoids and anthraquinones compared with the other extracts tested, experimental results did not confirm the expected antioxidant activity. It is possible that the different polarity of the extracts may contain different antioxidant components of variable reactivity as determined by the four in vitro models used in this work. Indeed, the DCM extract showed the most powerful DPPH radical capture activity, suggesting that compounds with the highest radical elimination activity in this species are of medium polarity. Furthermore, the extract’s free radical scavenging ability may be related to the presence and nature of contributing molecules’ electron transfer and hydrogen donating ability, including: intermedeol, which is an eudesmane-type sesquiterpene with an antiproliferative activity on promyelocytic leukemia HL-60 cells associated to both differentiation and apoptosis-inducing effects [18]; dehydrocostus lactone, which has been shown to present a powerful antioxidant activity that protects osteoblasts from antimycin A-induced cell damage via activation of PI3K/Akt/CREB [19]; and methyl pyruvate, which protects neuronal cells through its antioxidant actions on mitochondria [20]. It should also be noted that the level of antioxidants, as well as the synergistic effect that occurs between them and other plant constituents, can influence differences in the antioxidant capacity of plant extracts [21]. In this pathway, glutathione is the main low-molecular weight thiol in most plant cells [22], detected in mitochondria, chloroplasts, peroxisomes, apoplast, and vacuoles of different plant species [23] and isolatable from whole tissue extract [24]. Plants need glutathione or g-glutamylcysteine containing homologues to survive [25]. The roles of glutathione include xenobiotic detoxification, antioxidant biochemistry and redox homeostasis, under normal or stress situations, and it constitutes one of the principal cellular antioxidant systems with other antioxidant defenses (polyphenols, flavonoids, tocopherols and ascorbate) to scavenge reactive oxygen and nitrogen species [26]. In plant physiological conditions without stress, tissues such as leaves maintain measurable GSH:GSSG ratios of at least 20:1 [27]. In general, our results show a low GSH/GSSH ratio, because the flowers were cut and then dried, and so the mechanical damage to a plant induces a rapid release and activation of apoplastic peroxidases, and an oxidative burst of reactive oxygen species (ROS), exceeding the capacity of the antioxidant defense system [27,28] by the upregulation of peroxidase genes, glutathione being an electron donor to glutathione peroxidases in the reduction of hydroperoxides [29].

On the other hand, cancer, one of the leading causes of death in the world [30], arises from a stepwise accumulation of genetic or epigenetic changes that liberate neoplastic cells from the homeostatic regulation that controls normal cell proliferation [31]. While chemotherapy is one of the most common and effective treatments, its side effects are often severe, and so research and development into new antitumoral compounds are needed. This work provides data on the cytotoxic effect of *L. rivularis* flower extracts against different cancer cell lines. Our data indicate that hexane, dichloromethane and ethyl acetate treatments had cytotoxic activity with IC_50_ values between 3–9 µg/mL in cancer cell lines HT-29, PC-3 and MCF-7 and values higher than 100 µg/mL in HEK-293 cells. These results showed that the extracts had an SI between 10–30, indicating a high selectivity (SI > 3 is considered as a good value: see [32].

A previous study by the authors found that *Leptocarpha rivularis* DC contained a high percentage of sesquiterpene lactones (SQL), with leptocarpin as the major component in leave and stem extracts, and exhibited a significant cytotoxic activity against cancer cell lines with IC_50_ values of 4.5, 3.8 and 3.1 µM against PC-3, HT.29 and MCF-7 cell lines, respectively [5], and SI values between 2.9 and 4.9 [5,33]; no previous reports have provided complete descriptions of the flower extracts of *Leptocarpha rivularis* DC and their composition, antioxidant capacity and cytotoxic effect against cancer cell lines. Indeed, GC-MS analysis of flower extracts have shown the presence of sesquiterpene lactones and other compounds with antitumoral activity, such as dehydrocostus lactone, grosheimin, reynosin, deoxysericealactone, tetraneurin D, caryophyllene oxide, scopoletin, and thujone.

Next, dehydrocostus lactone, possesses various biological activities, including anti-inflammatory [34], antioxidant [35] and anticancer activities [36]. Research has associated the anticancer activities of dehydrocostus lactone to the inhibition of cancer cell proliferation and induction of cancer cell apoptosis [37], inhibition of migration and invasion and inhibition of angiogenesis [38]. The induction of apoptosis is associated with increased protein [37], a reduction in mitochondrial membrane potential [39] and the release of cytochrome C, triggering the intrinsic pathway of apoptosis and/or the activation of caspases [40,41,42]. Another, caryophyllene oxide, possesses strong anticancer properties [43,44]. reducing levels of procancer proteins and apoptosis inhibitors (bcl-2, bcl-xL, IAP-1, IAP-2) and increasing levels of those with proapoptotic properties [43,44]. Next, α-thujone and beta-thujone have been used for the treatment of a number of diseases due to antioxidant activity [45,46] and antitumorigenic effects [47,48]., which show a significant decrease in cell viability, internucleosomal DNA fragmentation, mitochondrial membrane permeability, an increase in ROS generation, and the release of cytochrome c and caspase-3 activation [46,47,48,49]. Another, reynosin, exerts a hepatoprotective effect against thioacetamide-induced apoptosis and hepatocellular DNA damage in primary mouse hepatocytes cultures and in vivo mouse models. In vivo experiments demonstrate levels of aspartate aminotransferase and alanine aminotransferase are decreased in reynosin-treated mice [50]. Additionally, biological studies in a rodent Parkinson’s disease model system demonstrate the neuroprotective effect of reynosin against dopamine-induced neuronal cell death [51]. Moreover, scopoletin, also present in extract, is a coumarin present in different species of plants that possess anticancer properties inducing cell cycle arrest and caspase-3 activation in the prostate cancer cell line [52], while inducing PARP cleavage and DNA fragmentation in promyeloleukemic HL-60 cells which lead to apoptosis [53]; and is able to enhance the neuronal defense system and protect the brain from rotenone-induced oxidative stress and Parkinson’s disease [54].

In addition to sesquiterpene lactones, triterpenes and triterpenoids were also present, and have been reported to have potent antineoplastic activity [55]. For example, α amyrin, present in the extracts of our study, has strong antiproliferative activity against A549 cancer cell line, with an IC_50_ value of 9.28 μg/mL. Lupeol (which was present in DCM extracts) and stigmasterol (AcOEt extracts) are major phytosterols in various herbal plants, possessing anti-inflammatory activities and candidates for anticancer agents. Studies demonstrate that lupeol and stigmasterol are anti-angiogenic compounds that inhibit endothelial cell proliferation, migration, and capillary network formation through the disruption of the TNF-α-VEGFR-2 axis, and they effectively suppress growth of cholangiocarcinoma xenografts by downregulating inflammatory cytokine production, macrophage recruitment and tumor angiogenesis [56]. Furthermore, lupeol has been shown to possess antioxidative [57], antiproliferative [58] and antitumor effects both in vitro and in vivo [59,60]. Specifically, lupeol was shown to possess significant antitumor activity in a two-stage model of mouse skin carcinogenesis, inducing cell cycle arrest and apoptosis in melanoma cell line 451Lu cells [61]. Moreover, lupeol has reduced the cell viability of HCT116 and SW620 colon cancer cell lines and can suppress the migration and invasion of colorectal cancer cells inhibiting the RhoA-ROCK1 pathway [62]. Lupeol and lupeol acetate have been shown to exhibit higher anti-inflammatory activity than the commonly used nonsteroidal anti-inflammatory drug indomethacin in rat and mouse models of inflammation [63]. Although lupeol has been shown to have anti-inflammation and antitumor capability, its poor bioavailability limits applications in living subjects. Lupeol acetate (LA), a derivative of lupeol, shows similar biological activities as lupeol but with better bioavailability [64]. Next, γ-sitosterol has been reported cytotoxic against colon and liver cancer cell lines, mediated by downregulation of c-myc expression and induction of the apoptotic pathways [65]. Another triterpenoid with anticancer and anti-inflammatory effects, betulinaldehyde [66] has been described as a retinoic acid receptor-related orphan receptor γt (RORγt) agonist [67], which alters the thermal stability of RORγt by directly binding to the protein in vitro, being a potential agent for tumor immunotherapy [68]. Another triterpenic compound present in *L. rivularis* is beta-Amyrin acetate. Plants with a high and majority beta-Amyrin acetate content show high antioxidant and cytotoxic activity [69]. Another compound present is linalool, an unsaturated terpene that exhibits antimicrobial, anti-inflammatory and antioxidant properties [70]. Moreover, linalool exhibits anticancer potential against prostate cancer, colon cancer, leukemia and cervical cancer [71,72,73]. It has also been shown to exert an inhibitory effect on cell proliferation of human solid tumors, including hepatocellular carcinoma, breast cancer, small cell carcinoma and malignant melanoma and induced apoptosis in both in vitro and in vivo models.

Other compounds present in *L. rivularis* flower extracts include retinoid “Fenretinide” (AcOEt extracts) (4-hydroxy (phenyl) (retinamide), previously described as a promising anticancer agent based on preclinical and clinical studies. Fenretinide has been established as cytotoxic to many kinds of cancer cells, and is reported to decrease cell viability and induce apoptosis in Ishikawa cells, an endometrial cancer cell line, dose dependently in vitro. Notably, this effect was found to be independent of the retinoic acid nuclear receptor signaling pathway, instead inducing apoptosis by increased retinol uptake via STRA6, which, when silenced, decreases apoptosis inhibited by knockdown of STRA6 expression in Ishikawa cells. [74]. Another compound found was grossheimin, a guaianolide with multiple biological activities: it is anti-inflammatory, inhibitory to inducible nitric oxide synthase; has antitumor and cytotoxic effects in VERO cell cultures, at 4.2 µg/L IC_50_ [75,76]. Moreover, grossheimin can decrease intracellular GSH in Jurkat cell line [76] and inhibit initial phases of TCR activation, having possible immunotherapeutic properties [77]. Finally, we detected thunbergol, also named isocembrol, a cembranoid that inhibits both α-glucosidase (IC_50_ 2.9 μg/mL) and NO production in activated macrophages (IC_50_ 3.6 μg/mL) [78]. Although we have discussed the cytotoxic effects of the individual principal components identified in Hex, DCM and AcOEt extracts, it is likely that there is some synergic effect among them that calls for further research.

## 4. Materials and Methods

### 4.1. General

All chemicals and solvents were obtained from Aldrich (St. Louis, MO, USA) and were used without further purification.

### 4.2. Plant Material

Flowers of *L. rivularis* DC were collected during the flowering season (November 2019) in Temuco (Región de la Araucania, Chile; 38°43′60″ S, 72°40′0″ W). A voucher specimen (No. Lr-11119) was deposited at the Herbarium of the Natural Products Laboratory, “LPNSO”, Department of Chemistry, Universidad de Playa Ancha, Valparaíso, Chile.

### 4.3. Extraction

The dried powdered flowers of *L. rivularis* DC (200 g) were exhaustively extracted with 70% ethanol (300 mL) by using Soxhlet extractor at 50 °C for 16 h. The polar extract was evaporated at low pressure to obtain crude ethanol extract which was then fractionated into hexane, dichloromethane, ethyl acetate and ethanol extracts [79]. All solvents used were chromatographic grade. Briefly, the extraction was carried out by using an orbital shaker (170 rpm) at 25 °C for 72 h. The resulting mixture was filtered through Whatman No. 1 filter paper (Sigma-Aldrich, Darmstadt, Germany) and the hexane was removed from the filtrate under reduced pressure with a rotatory evaporator (Rotavapor R-300, BÜCHI, Barcelona, Spain). The residue was further extracted with dichloromethane, ethyl acetate, and ethanol, sequentially and serially. Finally, each extract was weighed and the yield was calculated. *L. rivularis* DC flower extracts were kept at −4 °C prior to further analyses.

### 4.4. Phytochemical Screening of Flower Extracts

All flower extracts were subjected to qualitative chemical tests to identify various classes of bioactive chemical constituents present in the leaves using standard procedures [80].

#### 4.4.1. Determination of Total Phenols

Folin-Ciocalteu reagent was used to determine the total phenolic content (TPC) of the flower extracts [81]. Gallic acid was used as a reference standard (20–100 μg/mL) for plotting the calibration curve. A volume of 0.5 mL of the flower extract (100 μg/mL) was mixed with 1.5 mL of Folin-Ciocalteu reagent (diluted 1:10 with deionized water) and neutralized with 3 mL of sodium carbonate solution (7.5%, *w*/*v*). The reaction mixture was kept in the dark at room temperature for 30 min with intermittent shaking for color development. The absorbance of the resulting blue color was measured by using double beam UV-Vis spectrophotometer (UV Analyst-CT 8200) at a fixed wavelength of 765 nm. The TPCs were determined using the linear regression equation obtained from the standard plot of gallic acid. The content of total phenolic compounds was calculated as mean ± SD (*n* = 3) and expressed as mg/g gallic acid equivalent (GAE) of dry extract.

#### 4.4.2. Estimation of Total Flavonoid Content (TFC)

The TFC in the flower extracts was determined by the reported procedure of Madaan [82] and quercetin was used as a standard to construct the calibration curve. Briefly, 10 mg of quercetin was dissolved in 80% ethanol and then diluted to 20, 40, 60, 80 and 100 μg/mL. The diluted standard solutions of quercetin or plant extracts (0.5 mL) of different concentrations were separately mixed with 1.5 mL of 95% ethanol, 0.1 mL of 10% aluminum chloride, 0.1 mL of 1 mol/L potassium acetate and 2.8 mL of distilled water in a test tube. The test tubes were incubated for 30 min at room temperature to complete the reaction. The absorbance of the reaction mixture was measured at 415 nm with double beam UV-Vis spectrophotometer against blank. A typical blank solution contained all reagents except aluminum chloride which is replaced by the same amount of distilled water. The amount of flavonoid was calculated from the linear regression equation obtained from the quercetin calibration. All of the determinations were performed in triplicate.

#### 4.4.3. Total Anthraquinone Content (TAC)

TAC in the flower extracts was determined by the reported procedure [83,84] and emodin was used as a standard to construct the calibration curve. Briefly, 1 mL of 2% *w*/*v* aluminum trichloride (AlCl_3_) in ethanol was mixed with the same volume of the extract solution in ethanol (1.0 mg/mL). The mix was incubated for 10 min at room temperature, and absorbance was measured at 486 nm against a blank sample consisting of 1.0 mL extract solution with 1.0 mL of methanol without AlCl_3_. The total anthraquinone content was expressed as μg of emodin equivalents (EE)/g of dry extract. All of the determinations were performed in triplicate.

### 4.5. Chromatographic Analysis

The Hex, DCM and AcOEt extracts were diluted with chloroform and analysis by gas chromatography (Hewlett Packard, Palo Alto, CA, USA) was carried out according to the method detailed elsewhere [85,86]. The operating conditions were as follows: on-column injection; injector temperature, 250 °C; detector temperature, 280 °C; carrier gas, He at 1.0 mL/min; oven temperature program: 40 °C increase to 260 °C at 4 °C /min, and then 260 °C for 5 min, to afford the best separation through a capillary Rtx-5MS column. The mass detector ionization employed an electron impact of 70 eV. Compounds in the chromatograms were identified by comparison of their mass spectra with those in the NIST/EPA/NIH Mass spectral Library [87]. Chromatographic peaks were considered “unknown”, when their similarity index (MATCH) and reverse similarity index (RMATCH) were less than 850 and discarded in this identification process [88]. These parameters are referred to the degree the target spectrum matches the standard spectrum in the NIST Library (the value 1000 indicates a perfect fit), and by comparison of their retention index with those reported in the literature [87], for the same type of column or those of commercial standards, when available. The retention indices were determined under the same operating conditions in relation to a homologous n-alkanes series (C_8_–C_36_) by the equation:

RI = 100 × (n + Tr(unknown) − Tr(n)/Tr(N) − Tr(n)),(1)

where n = the number of carbon atoms in the smaller n-alkane; N = the number of carbon atoms in the larger nalkane; and Tr = the retention time. Component relative concentrations were obtained by peak area normalization.

### 4.6. Antioxidant Assay

#### 4.6.1. DPPH Radical Scavenging Assay

The DPPH assay was performed as described previously [89]. Briefly, 0.1 mL sample (from 0 to 4 mg/L of flower extracts) was mixed with 2.9 mL DPPH● solution (50 μM) and such a solution was freshly prepared in ethanol. A measure of 2.9 mL 50 μM DPPH● solution with 0.1 mL ethanol was used as a control. The absorbance of the resulting solutions, control and the blank (with the reagents only) were recorded after 15 min at room temperature. Each sample was replicated three times. The disappearance of DPPH● was detected spectrophotometrically at 517 nm. The percentage RSC (Radical Scavenging Capacity) was calculated by the following equation:

RSC (%) = 100% × (Acontrol − Asample)/Acontrol(2)

From the obtained RSC (%) values the IC_50_ value, which represent the concentrations of the resinous exudate and the major compounds that caused 50% inhibition, was determined by linear regression analysis.

#### 4.6.2. Ferric Reducing Antioxidant Potential (FRAP) Assay

The ferric reducing power of plant extracts was determined using the FRAP assay [90] with minor modifications. The working FRAP reagent was daily prepared by mixing 10 volumes of 300 mM acetate buffer, pH 3.6, with 1 volume of 10 mM TPTZ in 40 mM hydrochloric acid and with 1 volume of 20 mM ferric chloride. All solutions were used on the day of preparation. A sample of 100 µL of the extracts (1.0 mg/mL) in 300 μL of deionized water was added to 3 mL of freshly prepared FRAP reagent. The reaction mixture was incubated for 30 min at 37 °C in a water bath. Then, the absorbance of the samples was measured at 593 nm. An ethanol blank reading was also taken. The difference between sample absorbance and blank absorbance was calculated and used to determine the FRAP value. FRAP values were expressed as mM Trolox. All measurements were done in triplicate.

#### 4.6.3. Total Reactive Antioxidant Properties (TRAP) Assay

The assay was performed by the method set out previously [91]. A 10 mM solution of ABAP was mixed with 150 µM solution of ABTS•+ in 100 mM solution of PBS a pH 7.4. The mixture was incubated at 45 °C for 30 min. A measure of 10 µL of sample solution was added to 990 µL of the resulting blue-green ABTS•+ solution. The decrease of absorbance of TRAP solutions and ABTS•+ as blank were recorded after 30 s at room temperature. Then, the absorbance of the samples was measured at 734 nm. The total antioxidant capacity TRAP of the extracts was expressed in mM Trolox equivalents (TEAC), using a standard curve of Trolox (0–120 mg/L). All measurements were replicated three times.

#### 4.6.4. Oxygen Radical Absorbance Capacity (ORAC) Assay

Oxygen radical scavenging capacity was determined using the ORAC assay according to Alarcón [92]. ORAC-fluorescein is a convenient method that is extensively employed for the estimation of the antioxidant capacity of pure compounds or complex mixtures, such as herbal infusions and teas. The ORAC-index has previously been shown to depend on the target molecule used [93]. The ORAC values of the extracts were expressed as μM of Trolox equivalent antioxidant capacity (μM TEAC) based on a Trolox standard curve. Positive controls used were GA and BHT. All experiments were carried out in triplicate.

#### 4.6.5. Reduced Glutathione Assay

GSH was assayed by the enzymatic recycling procedure in which it is sequentially oxidized by 5,5’-dithiobis (2-nitrobenzoic acid) and reduced by NADPH in the presence of GR according to established methods [94,95] and adapted to *L. rivularis* DC flower extracts of the different solvent analyzed. Briefly, extraction was performed in 5% sulphosalicylic acid on g dry weight (DW) of *L. rivularis* DC flower extracts and the extent of 2-nitro-5- thiobenzoic acid formation was monitored at 412 nm for GSH plus GSSG evaluation. For determination of GSSG alone, the dry extract was pretreated with 2-vinylpyridine to scavenge GSH by derivatization.

### 4.7. Cell Viability

#### 4.7.1. Cell Culture

Human cancer cell lines: HT-29 (colon), MCF-7 (breast), PC-3 (prostate) and a nontumoral human cell line HEK-293 (embrionic kidney) were maintained in Ham’s-F12: DMEM high glucose medium (Gibco, San Diego, CA, USA), supplemented with 10% (*v*/*v*) fetal bovine serum (FBS) at 37 °C in a humidified 5% CO_2_ incubator. Appropriate volumes of the work solutions were added to the medium to reach the indicated concentrations (0, 0.625, 1.25, 2.5, 3.75, 6.25, 12.5, 25, 37.5 or 62.5 µg/mL) and cells were then incubated for the indicated periods of time.

#### 4.7.2. In Vitro Growth Inhibition Assay

The sulforhodamine B assay was used according to the method of Vichai and Kirtikara [96,97]. Test extracts were solubilized just prior to the experiment in 0.1% DMSO. Briefly, the cells were set up at 3 × 10^3^ cells per well of a 96-well, flat-bottomed 200 μL well microplate. Cells were incubated at 37 °C in a humidified 5% CO_2_/95% air mixture and treated with the extracts at different concentrations for 72 h. The cells which received only the medium containing 0.1% DMSO served as the control group. At the end of drug exposure, cells were fixed with 50% trichloroacetic acid at 4 °C. After washing with water, cells were stained with 0.1% sulforhodamine B (Sigma-Aldrich, St. Louis, MO, USA), dissolved in 1% acetic acid (50 μL/well) for 30 min, and subsequently washed with 1% acetic acid to remove unbound stain. Protein-bound stain was solubilized with 100 μL of 10 mM unbuffered Tris base, and the cell density was determined using a fluorescence plate reader (wavelength 540 nm). Leptocarpin was used at 9 μg/mL as a control positive. Values shown are the mean ± SD of three independent experiments in triplicate. The software used to calculate the IC_50_ values was SigmaPlot 11.0.

#### 4.7.3. Morphological Assessment of Cell Apoptosis

Cell morphology were analyzed, after 48 h of treatment to different extracts (37.5 µg/mL), using phase contrast microscopy. Briefly, HEK-293, HT-29, PC-3 and MCF-7 (1 × 10^4^ cells/mL) were cultured on 24-well chamber slides, and exposed to compounds for 48 h. The control group was exposed to 1% ethanol.

#### 4.7.4. Determination of Mitochondrial Membrane Permeability by Flow Cytometry

Rhodamine 123, a cationic voltage-sensitive probe that reversibly accumulates in mitochondria, was used to detect changes in mitochondrial membrane potential. Cells were incubated with different extracts (6.25 and 37.5 μg/mL) for 48 h, and subsequently were stained with rhodamine 123 (1 μM) and incubated in darkness for 1 h at 37 °C. Then, the medium was removed and cells were washed twice with PBS. Later the cells were trypsinized and collected by centrifugation (10 min at 1500 g). The supernatant was discarded and the cells pellets were resuspended in PBS and analyzed by flow cytometry using the filter FL1. Results are expressed as the percentage of cells stained with rhodamine 123.

#### 4.7.5. Determination of Caspases Activation

The activity of caspases was determined by using a fluorescent inhibitor of caspases tagged with fluorescein isothiocyanate, FITC-VAD-FMK. The CaspACE FITCVAD- FMK In Situ Marker was obtained from Promega. Briefly, cells were treated with extract (37.5 μg/mL) for 48 h. The cells were incubated with CaspACE FITC-VAD-FMK in darkness for 20 min at room temperature. Then, the medium was removed and cells were washed twice with PBS. Exposed cells were collected by tripsinization and centrifugation (10 min at 1500 g). The supernatant was discarded and the cells were resuspended in PBS and analyzed by flow cytometry using the filter FL3. Results are expressed as the percentage of cells stained with CaspACE FITC-VADFMK.

### 4.8. Statistical Analysis

Data are reported as mean values ± SD. Experiments were repeated three times, with triplicate samples for each. Data were analyzed by Prism 6 version 6.0d. Statistical significance was defined as *p* < 0.05. After tests of normality (Kolmogorov-Smirnov), one-way ANOVA followed by Tukey-Kramer multiple comparisons test as post hoc were applied, using 5% significance level.

## 5. Conclusions

Based on its potential for antioxidant and cytotoxic activities, *L. rivularis* DC flower extracts have been assessed for the first time. There seem to be positive correlations between antioxidant capacity and cell viability of cancer lines. Flower extracts demonstrated selective cytotoxicity and a caspase induction causing cell death, which suggests that *L. rivularis* DC flowers may be promising sources of natural antioxidants and other antitumoral or otherwise bioactive compounds for food and pharmaceutical industries.

## Figures and Tables

**Table 1:** Phytochemical contents of different flower extracts of *L. rivularis*.

Extract	Total Phenols (GAE mg·L^−1^)	S.D.	Total Flavonoids (QE mg·L^−1^)	S.D.	Total Anthraquinones (EE mg·L^−1^)	S.D.
**Hex**	27.8423 **a**	1.7642	70.9333 **a**	1.8986	37.8368 **a**	0.8442
**DCM**	41.8810 **b**	1.4569	26.4295 **b**	0.4815	10.4092 **b**	0.2330
**AcOEt**	47.8899 **b**	0.0449	33.8589 **b**	1.0233	12.9897 **b**	0.3568
**EtOH**	82.6696 **c**	5.8292	103.6202 **c**	3.9844	75.2023 **c**	1.7563

Hex: hexane; DCM: dichloromethane; AcOEt: ethyl acetate; EtOH: ethanol; GAE: gallic acid equivalent; QE: quercetin equivalents; EE: emodin equivalents; SD: standard deviation. Different letters in the same column indicate significant differences; *p* < 0.05, *n* = 3.

**Table 2:** Antioxidant activity of flower extracts of *L. rivularis*.

Extract	DPPH (IC_50_ mg·mL^−1^)	S.D.	FRAP (TEAC mM)	S.D.	TRAP (TEAC mM)	S.D.	ORAC (TEAC µM)	S.D
**Hex**	4.1863 **a**	0.1834	0.3141 **a**	0.0131	0.0074 **a**	0.0022	442.57 **a**	12.21
**DCM**	1.8953 **b**	0.0437	0.2656 **a**	0.0145	0.0961 **b**	0.0047	932.40 **b**	38.55
**AcOEt**	2.4088 **c**	0.0758	0.3063 **a**	0.0081	0.1288 **b**	0.0057	1878.17 **c**	37.35
**EtOH**	2.8300 **c**	0.0626	0.3687 **b**	0.0277	0.0729 **b**	0.0204	744.20 **d**	26.61
**T**	0.1060 **d**	0.0050	n.a.	n.a.	n.a.	n.a.	n.a	n.a
**GA**	n.a.	n.a.	1.7200 **c**	0.0200	1.1300 **c**	0.0100	696.22 **e**	63.84
**BHT**	0.0600 **d**	0.0010	1.5200 **d**	0.0700	1.0600 **c**	0.0200	593.87 **f**	41.27

Hex: hexane; DCM: dichloromethane; AcOEt: ethyl acetate; EtOH: ethanol; T: Trolox; GA: gallic acid; BHT: Butylhydroxytoluene; (2,2-diphenyl-1-picryl-hydrazyl-hydrate (DPPH), ferric reducing antioxidant potential (FRAP), total reactive antioxidant properties (TRAP) and oxygen radical absorbance capacity (ORAC)); IC_50_: represents the concentrations of extracts that caused the neutralization of 50% of the radical; TEAC: Trolox equivalent antioxidant capacity; SD: standard deviation. Different letters in the same column indicate significant differences; *p* < 0.05, *n* = 3; n.a.: not applicable.

**Table 3:** Glutathione pool of *L. rivularis* DC flower extracts.

Extract	GSH	GSSG	GSH/GSSG
	µmol/gDW		
**Hex**	1.179 ± 0.909 **d**	16.432 ± 3.756 **c**	0.062 ± 0.042 **b**
**DCM**	28.185 ± 3.385 **b**	22.110 ± 5.684 **b**	1.376 ± 0.204 **a**
**AcOEt**	11.461 ± 1.924 **c**	50.427 ± 5.686 **b**	0.224 ± 0.013 **b**
**EtOH**	86.456 ± 1.755 **a**	267.863 ± 9.157 **a**	0.323 ± 0.009 **b**

GSH: reduced glutathione; GSSG: oxidized glutathione; GSH/GSSG: ratio GSH/GSSG; gDW: grams dry weight of extract; DCM: dichloromethane; AcOEt: ethyl acetate; EtOH: ethanol. Different letters indicate significant differences (*p* < 0.05; Tukey test) ANOVA. Each value represents mean ± SE (*n* = 3).

**Table 4:** Composition of *n*-Hexane extract of *L. rivularis* flowers.

N° Peak	RT (min)	Main Components	RI ^a^	RIref ^b^	%Area ^c^	Match	Identification
1	8.94	Caryophyllene oxide	1534	1534	0.37	940	RI, MS
2	10.01	Elemol	1539	1537	0.11	900	RI, MS
3	10.41	Isoaromadendrene epoxide	1580	1579	0.04	890	RI, MS
4	11.86	7-Hydroxyfarnesen	1582	1584	2.81	870	RI, MS
5	12.15	Paradisol	1624	1627	1.33	940	RI, MS
6	12.31	trans-*Z*-a-Bisabolene epoxide	1823	1820	0.31	850	RI, MS
7	13.64	Dehydrocostus lactone	1865	1866	0.06	870	RI, MS
8	16.86	2-methyl-1-hexadecanol	1888	1890	0.29	880	RI, MS
9	17.51	Scopoletin	1924	1924	0.90	870	RI, MS, Co
10	18.32	Tetradecanoic acid	1940	1940	0.04	920	RI, MS
11	22.07	Eicosane	2000	2000	0.63	900	RI, MS
12	22.24	α-Linolenic acid	2100	2102	0.27	920	RI, MS
13	22.81	Grosheimin	2143	2143	1.58	850	RI, MS, Co
14	23.28	(*Z*)-18-Octadec-9-enolide	2155	2158	0.52	950	RI, MS
15	25.67	Glyceryl linolenate	2157	2161	0.05	880	RI, MS
16	26.46	Reynosin	2266	2266	1.35	900	RI, MS, Co
17	31.66	4,8,13-Duvatriene-1,3-diol	2397	2400	25.70	860	RI, MS
18	34.62	3-Ethyl-5-(2-ethylbutyl)octadecane	2410	2413	3.89	930	RI, MS
19	34.81	Tetraneurin D	2490	2494	0.69	970	RI, MS
20	35.62	2-Methylenecholestan-3-ol	2650	2652	0.41	970	RI, MS
21	35.86	*α*-Tocopherol	3149	3149	0.35	950	RI, MS, Co
22	37.69	Stigmasterol	3170	3170	0.70	940	RI, MS, Co
23	40.50	γ-Sitosterol	3290	3290	0.33	930	RI, MS, Co
24	42.69	ß-Amyrin	3330	3337	0.35	850	RI, MS
25	43.79	ß -Amyrin acetate	3333	3339	4.78	910	RI, MS
26	44.57	Lupeyl acetate	3380	3380	10.96	850	RI, MS, Co
27	46.06	Betulinaldehyde	3630	3629	0.13	820	RI, MS
28	46.62	1-Heptatriacotanol	3941	3942	0.46	900	RI, MS
		Total identified			59.41		
		Unknown			40.59		

^a^ RI: retention indices relative to C_8_-C_36_ *n*-alkanes on the HP-5 MS capillary column; ^b^ RI: retention index from the literature [9]; ^c^ surface area of GC peak; MS: comparison of the mass spectra with those of the NIST 14; Co: co-elution with standard compounds available in our laboratory.

**Table 5:** Composition of DCM extract of *L. rivularis* flowers.

N° Peak	RT (min)	Main Components	RI ^a^	RIref ^b^	%Area ^c^	Match	Identification
1	8.92	pyrulic acid methyl ester	1210	1217	2.50	880	RI, MS
2	9.98	10-epi-Elemol	1549	1550	0.53	900	RI, MS
3	10.39	Cariophyllene oxide	1600	1606	0.24	910	RI, MS
4	12.13	Intermedeol	1660	1675	2.94	810	RI, MS
5	12.66	Tetradecanoic acid	1765	1770	0.07	890	RI, MS
6	13.63	Tetradecanoic acid, ethyl ester	1783	1790	0.55	930	RI MS
7	14.09	Deoxyserecialactone	1825	NF	0.30	900	RI, MS
8	14.25	Neophytadiene	1835	1836	0.15	870	RI, MS
9	15.91	6,10,14-Trimethyl-2-pentadecanone	1852	1855	0.20	900	RI, MS
10	16.03	Dehydrocostus lactone	1860	1866	2.76	890	RI, MS, Co
11	22.01	9-trans-Methyl Trisporate C	2109	2100	0.51	890	RI, MS, Co
12	22.51	Glyceryl linolenate	2174	2160	0.06	860	RI, MS
13	23.25	5α,6α-Epoxy-3β,17-dihydroxy-16α-methylpregnan-20-one	2189	2189	0.71	850	RI, MS
14	25.66	(Z)-18-Octadec-9-enolide	2190	2208	2.57	980	RI, MS
15	26.48	Reynosin	2260	2266	1.05	860	RI, MS, Co
16	29.10	9-Tricosene, (Z)	2270	2299	0.80	870	RI, MS
17	29.90	Tricosane	2330	2300	3.85	970	RI, MS
18	31.68	2-[(Z)-9-Octadecenyloxy]etanol	2330	2336	0.36	870	RI, MS
19	32.67	3-Ethyl-5-(2’-ethylbutyl)octadecane	2410	2413	0.34	940	RI, MS
20	33.95	Thunbergol	2500	2498	8.82	940	RI, MS
21	23.18	2-Methylenecholestan-3-ol	2650	2652	1.15	920	RI, MS
22	36.05	Triacontane	3000	3000	4.36	870	RI, MS
23	36.34	ß-Amyrin	3315	3337	0.43	940	RI, MS
24	36.96	α-Amyrin	3360	3376	0.98	940	RI, MS
25	37.59	Tetratriacontane	3400	3400	6.39	860	RI, MS, Co
27	44.48	ß -amyrin acetate	3430	3437	6.43	910	RI, MS
28	44.59	Lupeol	3494	3486	0.25	930	RI, MS, Co
29	45.53	lupeyl acetate	3530	3525	14.96	900	RI, MS
30	45.67	Betulinaldehyde	3743	3760.	0.70	900	RI, MS
31	46.62	1-Heptatriacotanol	3940	3942	6.71	880	RI, MS, Co
32	50.52	Oleic acid 3-(octadecyloxy)propyl ester	4100	4149	0.76	850	RI, MS, Co
		Total identified			72.43		
		Unknown			27.57		

^a^ RI: retention indices relative to C_8_-C_36_ *n*-alkanes on the HP-5 MS capillary column; ^b^ RI: retention index from the literature [9]; ^c^ surface area of GC peak; MS: comparison of the mass spectra with those of the NIST 14; Co: co-elution with standard compounds available in our laboratory.

**Table 6:** Composition of AcOEt extract of *L. rivularis* flowers.

No.	RT (min)	Main Components	RI ^a^	RIref ^b^	% Area ^c^	Match	Identification
1	9.575	Thujone	1095	1089	6.46	927	RI, MS, Co
2	12.750	Isopentenyl acetate	1196	1200	2.87	950	RI, MS
3	13.215	*trans*-Sabinyl acetate	1265	1264	4.01	934	RI, MS
4	18.110	Intermedeol	1660	1667	3.44	949	RI, MS
5	18.234	6-*epi*-Shyobunol	1883	1881	3.59	953	RI, MS
6	18.450	Eicosane	2000	2000	2.87	989	RI, MS
7	18.610	Geranyllinalool	2040	2046	2.08	910	RI, MS
8	19.85	Leptocarpin	2050	2050	3.46	967	RI, Co
9	20.120	4,8,13-duvatriene-1,3-diol	2395	2400	6.20	960	RI, MS
10	21.250	Linalool	2486	2491	2.26	899	RI, MS, Co
11	21.590	2-Methylenecholestan-3-ol	2651	2652	2.32	956	RI, MS
12	42.06	Stigmasterol	3140	3142	24.02	940	RI, MS, Co
13	42.70	Fenretinide	3290	3289	13.76	930	RI, MS, Co
14	43.86	1-Heptatriacotanol	3938	3942	8.49	900	RI, MS
		Total identified			85.83		
		Unknown			14.17		

^a^ RI: retention indices relative to C_8_-C_36_ *n*-alkanes on the HP-5 MS capillary column; ^b^ RI: retention index from the literature [9]; ^c^ surface area of GC peak; MS: comparison of the mass spectra with those of the NIST 14; Co: co-elution with standard compounds available in our laboratory.

**Table 7:** IC_50_ values (µg/mL) of *L. rivularis* flower extracts against different cancer cell lines (PC-3, HT-29 and MCF-7) and HEK-293 (human embryonic kidney cell line).

Extracts	PC-3	HT-29	MCF-7	HEK-293
**Hex**	4.49 ± 0.54 *	3.94 ± 0.52 *	4.71 ± 0.60 *	82.94 ± 9.59
**DCM**	8.46 ± 4.53 *	3.04 ± 0.79 *	4.61 ± 1.47 *	>100
**AcOEt**	8.80 ± 3.04 *	3.69 ± 0.38 *	4.46 ± 0.98 *	>100
**EtOH**	90.95 ± 31.5	179.5 ± 18.3	214.2 ± 54.7	>100
**Leptocarpin**	1.80 ± 0.31	1.43 ± 0.25	1.28 ± 0.21	6.28 ± 0.53

Data are reported as mean values ± SD of three different experiments with samples in triplicate. * *p* < 0.05; significantly different from the ethanolic extract-treated cells.

**Table 8:** Selectivity Index (SI) values of flower extracts of *Leptocarpha rivularis* DC for different cancer cell lines (PC-3, HT-29 and MCF-7).

Extracts	PC-3	HT-29	MCF-7
**Hex**	21.05	18.47	17.60
**DCM**	>32.89	>11.82	>21.61
**AcOEt**	>27.10	>11.36	>22.42
**EtOH**	>0.55	>1.01	>0.47
**Leptocarpin**	3.48	4.39	4.90

**Table 9:** Percentage of rhodamine 123-stained cells after treatment with different extracts on HT-29, MCF-7, PC-3 and HEK-293 cell lines.

Extracts	Concentration (µg/mL)	HT-29	MCF-7	PC-3	HEK-293
****Hex****	6.25	81.3 ± 7.3	74.6 ± 8.2	82.3 ± 6.9	78.3 ± 5.8
	37.5	35.7 ± 5.4 *	44.9 ± 6.1 *	57.5 ± 6.7 *	73.5 ± 7.2
****DCM****	6.25	36.3 ± 5.9 *	44.6 ± 5.1 *	53.2 ± 5.4 *	67.2 ± 10.4 *
	37.5	9.3 ± 1.6 *	11.9 ± 5.0 *	17.4 ± 6.2 *	61.4 ± 8.2 *
****AcOEt****	6.25	81.7 ± 8.7	76.3 ± 9.1	69.9 ± 6.3 *	77.9 ± 8.3
	37.5	13.3 ± 1.4 *	23.5 ± 5.0 *	26.2 ± 4.3 *	69.2 ± 6.4 *
****Leptocarpin****	9.0 (25 µM)	53.3 ± 5.6 *	21.5 ± 4.2 *	46.5 ± 7.9 *	76.9 ± 8.7
****Control (ethanol**)**	-	92.0 ± 10.2	81.4 ± 8.3	84.5 ± 7.5	80.8 ± 8.7

Values are mean ± S.D. (*n* = 3); * *p* < 0.05; significantly different from the control-(ethanol) treated cells.

## Data Availability

All data are availables for cientific community.

## Sample Availability

Samples of the extracts are available from the authors.

## References

[1] Riedemann P., Aldunate G. (2011). Flora Nativa de Valor Ornamental. Identificación y Propagación. Chile—Zona Sur.

[2] Urban O. (1934). Botánica de las Plantas Endémicas de Chile.

[3] Hoffman A. (1982). Flora Silvestre de Chile. Zona Austral.

[4] Martinez R., Kesternich V., Carrasco H., Alvarez-Contreras C., Montenegro C., Ugarte R., Gutierrez E., Moreno J., Garcia C., Werner E. (2006). Synthesis and conformational analysis of leptocarpin derivatives. Influence of modification of the oxirane ring onleptocarpin’s cytotoxic activity. J. Chil. Chem. Soc..

[5] Bosio C., Tomasoni G., Martínez R., Olea A.F., Carrasco H., Villena J. (2015). Cytotoxic and apoptotic effects of leptocarpin, a plant-derived sesquiterpene lactone, on human cancer cell lines. Chem. Biol. Interact..

[6] Mena C. (2008). Efecto apoptótico y quimiosensibilizador de leptocarpina en distintos modelos celulares de leucemias, E. A. Y. Q. *O: Medicina*. Undergraduate Thesis.

[7] Alvarez C. (2005). Efecto hipoglucemiante de la infusión de *Leptocarpha rivularis* en ratas sprague-dawley diabéticas tipo ii por inducción con aloxano. Undergraduate Thesis.

[8] Montenegro I., Ramirez I., Dorta F., Madrid A., Seeger M. Micropropagation and determination of the antioxidant capacity of the *Leptocarpha rivularis*. Proceedings of the X Plant Biology Meeting.

[9] Adams R.P. (2007). Identification of Essential Oil Components by Gas Chromatography/Mass Spectrometry.

[10] Zamzami N., Larochette N., Kroemer G. (2005). Mitochondrial permeability transition in apoptosis and necrosis. Cell Death Differ..

[11] Obrenovich M.E., Li Y., Parvathaneni K., Yendluri B.B., Palacios H.H., Leszek J., Aliev G. (2011). Antioxidants in health, disease and aging. CNS Neurol. Disord. Drug Targets.

[12] Diaz-Munoz G., Miranda I.L., Sartori S.K., de Rezende D.C., Diaz M.A. (2018). Anthraquinones: An Overview. Studies in Natural Products Chemistry.

[13] Mishra A., Sharma A.K., Kumar S., Saxena A.K., Pandey A.K. (2013). Bauhinia variegate leaf extracts exhibit considerable antibacterial, antioxidant, and anticancer activities. Biomed. Res. Int..

[14] Brusselmans K., De Schrijver E., Heyns W., Verhoeven G., Swinnen J.V. (2003). Epigallocatechin-3-gallate is a potent natural inhibitor of fatty acid synthase in intact cells and selectively induces apoptosis in prostate cancer cells. Int. J. Cancer.

[15] Brusselmans K., Vrolix R., Verhoeven G., Swinnen J.V. (2005). Induction of cancer cell apoptosis by flavonoids is associated with their ability to inhibit fatty acid synthase activity. J. Biol. Chem..

[16] Kumar S., Pandey A.K. (2013). Chemistry and biological activities of flavonoids: An overview. Sci. World J..

[17] Jarial R., Thakur S., Sakinah M., Zularisam A.W., Sharad A., Kanwar S.S., Singh L. (2018). Potent anticancer, antioxidant and antibacterial activities of isolated flavonoids from *Asplenium nidus*. J. King Saud Univ. Sci..

[18] Jeong S.H., Koo S.J., Choi J.H., Park J.H., Ha J., Park H.J., Lee K.T. (2002). Intermedeol isolated from the leaves of *Ligularia fischeri* var. spiciformis induces the differentiation of human acute promyeocytic leukemia HL-60 cells. Planta Med..

[19] Seo M.S., Choi E.M. (2012). The effects of dehydrocostus lactone on osteoblastic MC3T3-E1 cells in redox changes and PI3K/Akt/CREB. Immunopharmacol. Immunotoxicol..

[20] Wang X., Perez E., Liu R., Yan L.J., Mallet R.T., Yang S.H. (2007). Pyruvate protects mitochondria from oxidative stress in human neuroblastoma SK-N-SH cells. Brain Res..

[21] Xu X., Li F., Zhang X., Li P., Zhang X., Wu Z., Li D. (2014). In vitro synergistic antioxidant activity and identification of antioxidant components from *Astragalus membranaceus* and *Paeonia lactiflora*. PLoS ONE.

[22] Herschbach C., Scheerer U., Rennenberg H. (2010). Redox states of glutathione and ascorbate in root tips of poplar (Populus tremula×P. alba) depend on phloem transport from the shoot to the roots. J. Exp. Bot..

[23] Zechmann B., Müller M. (2010). Subcellular compartmentation of glutathione in dicotyledonous plants. Protoplasma.

[24] Vanacker H., Carver T.L.W., Foyer C.H. (1998). Pathogeninduced changes in the antioxidant status of the apoplast in barley leaves. Plant Physiol..

[25] Cairns N.G., Pasternak M., Wachter A., Cobbett C.S., Meyer A.J. (2006). Maturation of Arabidopsis seeds is dependent on glutathione biosynthesis within the embryo. Plant Physiol..

[26] de Sousa A., AbdElgawad H., Asard H., Pinto A., Soares C., Branco-Neves S., Braga T., Azenha M., Selim S., Al Jaouni S. (2017). Metalaxyl Effects on Antioxidant Defenses in Leaves and Roots of *Solanum nigrum* L.. Front. Plant Sci..

[27] Noctor G., Mhamdi A., Chaouch S., Han Y., Neukermans J., Marquez-Garcia B., Queval G., Foyer C. (2012). Glutathione in plants: An integrated overview. Plant Cell Environ..

[28] Hasanuzzaman M., Bhuyan M., Anee T.I., Parvin K., Nahar K., Mahmud J.A., Fujita M. (2019). Regulation of Ascorbate-Glutathione Pathway in Mitigating Oxidative Damage in Plants under Abiotic Stress. Antioxidants.

[29] Minivayeva F., Beckett R., Kranner I. (2015). Roles of apoplastic peroxidases in plant response to wounding. Phytochemistry.

[30] Bray F., Ferlay J., Soerjomataram I., Siegel R.L., Torre L.A., Jemal A. (2018). Global cancer statistics 2018: GLOBOCAN estimates of incidence and mortality worldwide for 36 cancers in 185 countries. CA Cancer J Clin..

[31] Hanahan D., Weinberg R.A. (2011). Hallmarks of cancer: The next generation. Cell.

[32] De Oliveira P., Alves J., Damasceno J., Oliveira R., Dias H., Crotti A., Tavares D. (2015). Cytotoxicity screening of essential oils in cancer cell lines. Rev. Bras. Farmacogn..

[33] Martinez R., Kesternich V., Gutierrez E., Dolz H., Mansilla H. (1995). Conformational-Analysis and biological activity of leptocarpin and leptocarpin acetate. Planta Med..

[34] Butturini E., di Paola R., Suzuki H., Paterniti I., Ahmad A., Mariotto S., Cuzzocrea S. (2014). Costunolide and dehydrocostuslactone, two natural sesquiterpene lactones, ameliorate the inflammatory process associated to experimental pleurisy in mice. Eur. J. Pharmacol..

[35] Eliza J., Daisy P., Ignacimuthu S. (2010). Antioxidant activity of costunolide and eremanthin isolated from Costus speciosus (Koen ex. Retz) Sm. Chem. Biol. Interact..

[36] Kuo P.L., Ni W.C., Tsai E.M., Hsu Y.L. (2009). Dehydrocostuslactone disrupts signal transducers and activators of transcription 3 through up-regulation of suppressor of cytokine signaling in breast cancer cells. Mol. Cancer Ther..

[37] Choi E.J., Ahn W.S. (2009). Antiproliferative effects of dehydrocostuslactone through cell cycle arrest and apoptosis in human ovarian cancer SK-OV-3 cells. Int. J. Mol. Med..

[38] Hao L.J., Zhao F., Gao Z.T., Xu H., Liu K. (2010). Inhibitory efects of sesquiterpenes from *Saussurea lappa* on the vascular endothelial growth factor. Nat. Prod. Res. Dev..

[39] Long H.Y., Huang Q.X., Yu Y.Y., Zhang Z.B., Yao Z.W., Chen H.B., Feng J.W. (2019). Dehydrocostus lactone inhibits in vitro gastrinoma cancer cell growth through apoptosis induction, sub-G1 cell cycle arrest, DNA damage and loss of mitochondrial membrane potential. Arch. Med. Sci..

[40] Kim E.J., Hong J.E., Lim S.S., Kwon G.T., Kim J., Kim J.S., Lee K.W., Park J.H. (2012). The hexane extract of *Saussurea lappa* and its active principle, dehydrocostus lactone, inhibit prostate cancer cell migration. J. Med. Food.

[41] Kim E.J., Lim S.S., Park S.Y., Shin H.K., Kim J.S., Yoon Park J.H. (2008). Apoptosis of DU145 human prostate cancer cells induced by dehydrocostus lactone isolated from the root of *Saussurea lappa*. Food Chem. Toxicol..

[42] Oh G.S., Pae H.O., Chung H.T., Kwon J.W., Lee J.H., Kwon T.O., Kwon S.Y., Chon B.H., Yun Y.G. (2004). Dehydrocostus lactone enhances tumor necrosis Factor-a-Induced apoptosis of human leukemia HL-60 cells. Immunopharmacol. Immunotoxicol..

[43] Jun N.J., Mosaddik A., Moon J.Y., Jang K.C., Lee D.S., Ahn K.S., Cho S.K. (2011). Cytotoxic activity of β-caryophyllene oxide isolated from Jeju Guava (*Psidium cattleianum* Sabine) leaf. Rec. Nat. Prod..

[44] Park K.R., Nam D., Yun H.M., Lee S.G., Jang H.J., Sethi G., Cho S.K., Ahn K.S. (2011). β-Caryophyllene oxide inhibits growth and induces apoptosis through the suppression of PI3K/AKT/mTOR/S6K1 pathways and ROS-mediated MAPKs activation. Cancer Lett..

[45] Naser B., Bodinet C., Tegtmeier M., Lindequist U. (2005). *Thuja occidentalis* (Arbor vitae): A review of its pharmaceutical, pharmacological and clinical properties. Evid. Based Complement. Alternat. Med..

[46] Dubey S.K., Batra A. (2009). Antioxidant activity of *Thuja occidentalis* linn. Asian J. Pharm. Clin. Res..

[47] Mukherjee A., Sikdar S., Bishayee K., Paul A., Ghosh S., Boujedaini N., Khuda-Bukhsh A.R. (2012). Ethanolic extract of *Thuja occidentalis* blocks proliferation of A549 cells and induces apoptosis in vitro. J. Chin. Integr. Med..

[48] Biswas R., Mandal S.K., Dutta S., Bhattacharyya S.S., Boujedaini N., Khuda-Bukhsh A.R. (2011). Thujone-rich fraction of *Thuja occidentalis* demonstrates major anti-cancer potentials: Evidences from in vitro studies on A375 cells. Evid. Based Complement. Alternat. Med..

[49] Torres A., Vargas Y., Uribe D., Carrasco C., Torres C., Rocha R., Oyarzún C., San Martín R., Quezada C. (2016). Pro-apoptotic and Anti-Angiogenic Properties of the α/β-Thujone Fraction from *Thuja Occidentalis* on Glioblastoma Cells. J. Neurooncol..

[50] Lim S., Lee S., Nam K., Kim K., Mar W. (2013). Hepatoprotective effects of reynosin against thioacetamide-induced apoptosis in primary hepatocytes and mouse liver. Arch. Pharm. Res..

[51] Ham A., Kim D., Kim K., Lee S., Oh K., Shin J., Mar W. (2013). Reynosin protects against neuronal toxicity in dopamine-induced SH-SY5Y cells and 6-hydroxydopamine-lesioned rats as models of Parkinson’s disease: Reciprocal up-regulation of E6-AP and down-regulation of α-synuclein. Brain Res..

[52] Moon P.D., Lee B.H., Jeong H.J., An H.J., Park S.J., Kim H.R., Ko S.G., Um J.Y., Hong S.H., Kim H.M. (2007). Use of scopoletin to inhibit the production of inflammatory cytokines through inhibition of the IκB/NF-κB signal cascade in the human mast cell line HMC-1. Eur. J. Pharmacol..

[53] Kim E., Kwon K., Shin B., Seo E., Lee Y., Kim J., Ryu D. (2005). Scopoletin induces apoptosis in human promyeloleukemic cells, accompanied by activations of nuclear factor κB and caspase-3. Life Sci..

[54] Narasimhan K., Jayakumar D., Velusamy P., Srinivasan A., Mohan T., Ravi D.B., Uthamaraman S., Sathyamoorthy Y.K., Rajasekaran N.S., Periandavan K. (2019). Morinda citrifolia and Its Active Principle Scopoletin Mitigate Protein Aggregation and Neuronal Apoptosis through Augmenting the DJ-1/Nrf2/ARE Signaling Pathway. Oxidative Med. Cell. Longev..

[55] Peron G., Marzaro G., Dall’Acqua S. (2018). Known triterpenes and their derivatives as scaffolds for the development of new therapeutic agents for cancer. Curr. Med. Chem..

[56] Kangsamaksin T., Chaithongyot S., Wootthichairangsan C., Hanchaina R., Tangshewinsirikul C., Svasti J. (2017). Lupeol and stigmasterol suppress tumor angiogenesis and inhibit cholangiocarcinoma growth in mice via downregulation of tumor necrosis factor-α. PLoS ONE.

[57] Nagaraj M., Sunitha S., Varalakshmi P. (2000). Effect of Lupeol, a pentacyclic triterpene, on the lipid peroxidation and antioxidant status in rat kidney after chronic cadmiumexposure. J. Appl. Toxicol..

[58] Laszczyk M., Jäger S., Simon-Haarhaus B., Scheffler A., Schempp C.M. (2006). Physical, chemical and pharmacological characterization of a new oleogel-forming triterpene extract from the outer bark of birch (*Betulae Cortex*). Planta Med..

[59] Lee T.K., Poon R.T., Wo J.Y., Ma S., Guan X.Y., Myers J.N., Altevogt P., Yuen A.P. (2007). Lupeol suppresses cisplatin-induced nuclear factor- KB activation in head and neck squamous cell carcinoma and inhibits local invasion and nodal metastasis in an orthotopic nude mouse model. Cancer Res..

[60] Saleem M., Kweon M.-H., Yun J.-M., Adhami V.M., Khan N., Syed D.N., Mukhtar H. (2005). A novel dietary triterpene Lupeol induces fas-mediated apoptotic death of androgen-sensitive prostate cancer cells and inhibits tumor growth in a xenograft model. Cancer Res..

[61] Saleem M., Afaq F., Adhami V.M., Mukhtar H. (2004). Lupeol modulates NF-κB and PI3K/Akt pathways and inhibits skin cancer in CD-1 mice. Oncogene.

[62] Jiang Y., Hong D., Lou Z., Tu X., Jin L. (2020). Lupeol inhibits migration and invasion of colorectal cancer cells by suppressing RhoA-ROCK1 signaling pathway. Naunyn Schmiedebergs Arch. Pharmacol..

[63] Saleem M. (2009). Lupeol, a novel anti-inflammatory and anti-cancer dietary triterpene. Cancer Lett..

[64] Wang W.H., Chuang H.Y., Chen C.H., Chen W.K., Hwang J.J. (2016). Lupeol acetate ameliorates collagen-induced arthritis and osteoclastogenesis of mice through improvement of microenvironment. Biomed. Pharmacother..

[65] Endrini S., Rahmat A., Ismail P., Taufiq-Yap Y.H. (2014). Cytotoxic effect of γ-sitosterol from Kejibeling (*Strobilanthes crispus*) and its mechanism of action towards c-myc gene expression and apoptotic pathway. Med. J. Indones..

[66] Gao Q.-H., Wu C.-S., Wang M. (2013). The jujube (*Ziziphus Jujuba* Mill.) fruit: A review of current knowledge of fruit composition and health benefits. J. Agric. Food Chem..

[67] Yang F., Zhang R., Ni D., Luo X., Chen S., Luo C., Xiao W. (2019). Discovery of betulinaldehyde as a natural RORγt agonist. Fitoterapia.

[68] Park J.S., Moon S.J., Lim M., Byun J.K., Hwang S.H., Yang S., Kim E.K., Kim S.M., Lee J., Kwok S. (2019). Retinoic Acid Receptor-Related Receptor Alpha Ameliorates Autoimmune Arthritis via Inhibiting of Th17 Cells and Osteoclastogenesis. Front. Immunol..

[69] Fabiyi O.A., Atolani O., Adeyemi O.S., Olatunji G.A. (2012). Antioxidant and cytotoxicity of β-amyrin acetate fraction from *Bridelia ferruginea* leaves. Asian Pac. J. Trop. Biomed..

[70] Zengin H., Baysal A.H. (2014). Antibacterial and antioxidant activity of essential oil terpenes against pathogenic and spoilage-forming bacteria and cell structure-activity relationships evaluated by SEM microscopy. Molecules.

[71] Iwasaki K., Zheng Y.W., Murata S., Ito H., Nakayama K., Kurokawa T., Sano N., Nowatari T., Villareal M.O., Nagano Y.N. (2016). Anticancer effect of linalool via cancer-specific hydroxyl radical generation in human colon cancer. World J. Gastroenterol..

[72] Chang M.Y., Shieh D.E., Chen C.C., Yeh C.S., Dong H.P. (2015). Linalool induces cell cycle arrest and apoptosis in leukemia cells and cervical cancer cells through CDKIs. Int. J. Mol. Sci..

[73] Zhao Y., Cheng X., Wang G., Liao Y., Qing C. (2020). Linalool inhibits 22Rv1 prostate cancer cell proliferation and induces apoptosis. Oncol. Lett..

[74] Mittal N., Malpani S., Dyson M., Ono M., Coon J.S., Kim J.J., Schink J.C., Bulun S.E., Pavone M.E. (2014). Fenretinide: A novel treatment for endometrial cancer. PLoS ONE.

[75] Bruno M., Bancheva S., Rosselli S., Maggio A. (2013). Sesquiterpenoids in subtribe Centaureinae (Cass.) Dumort (tribe Cardueae, Asteraceae): Distribution, 13C NMR spectral data and biological properties. Phytochemistry.

[76] Picman A. (1986). Biological activities of sesquiterpene lactones. Biochem. Syst. Ecol..

[77] Schepetkin I.A., Kirpotina L.N., Mitchell P.T., Kishkentaeva A.S., Shaimerdenova Z.R., Atazhanova G.A., Adekenov S.M., Quinn M.T. (2018). The natural natural sesquiterpene lactones arglabin, grosheimin, agracin, parthenolide, and estafiatin inhibit T cell receptor (TCR) activation. Phytochemistry.

[78] Shpatov A.V., Popov S.A., Salnikova O.I., Kukina T.P., Shmidt E.N., Um B.H. (2017). Composition and Bioactivity of Lipophilic Metabolites from Needles and Twigs of Korean and Siberian Pines (Pinus koraiensis Siebold & Zucc. and Pinus sibirica Du Tour). Chem. Biodivers..

[79] De Castro M.L., Garcıa-Ayuso L.E. (1998). Soxhlet extraction of solid materials: An outdated technique with a promising innovative future. Anal. Chim. Acta.

[80] Mohammad N., Sajid A., Muhammad Q. (2011). Preliminary phytochemical screening of flowers, leaves, bark, stem and roots of Rhododendron arboreum. Middle East J. Sci. Res..

[81] Aryal S., Baniya M.K., Danekhu K., Kunwar P., Gurung R., Koirala N. (2019). Total phenolic content, flavonoid content and antioxidant potential of wild vegetables from Western Nepal. Plants.

[82] Madaan R., Bansal G., Kumar S., Sharma A. (2011). Estimation of total phenols and flavonoids in extracts of Actaea spicata roots and antioxidant activity studies. Indian J. Pharm. Sci..

[83] Mellado M., Madrid A., Pena-Cortes H., López R., Jara C., Espinoza L. (2013). Antioxidant activity of anthraquinones isolated from leaves of Muehlenbeckia hastulata (je sm.) johnst.(polygonaceae). J. Chil. Chem. Soc..

[84] Kurkin V.A., Shmygareva A.A., Ryazanova T.K., San’kov A.N. (2017). Quantitative Determination of Total Anthraquinone Glycosides in Cassia Syrup Preparation. Pharm. Chem. J..

[85] Montenegro I., Madrid A., Zaror L., Martínez R., Werner E., Carrasco H., Cuellar M., Palma H. (2012). Antimicrobial activity of ethyl acetate extract and essential oil from bark of *Laurelia sempervirens* against multiresistant bacteria. Bol. Latinoam. Caribe Plant. Med. Aromat..

[86] Canales N., Montenegro I., Párraga M., Olguín Y., Godoy P., Werner E., Madrid A. (2016). In vitro antimicrobial activity of embothrium coccineum used as traditional medicine in patagonia against multiresistant bacteria. Molecules.

[87] NIST/EPA/NIH Mass Spectral Library with Search Program (Data Version: NIST 11, Software Version 2.0 g. http://webbook.nist.gov/chemistry/name-ser.html.

[88] Santander R., Creixell W., Sánchez E., Tomic G., Silva J.R., Acevedo C.A. (2013). Recognizing Age at Slaughter of Cattle from Beef Samples Using GC/MS-SPME Chromatographic Method. Food Bioprocess Technol..

[89] Leyton M., Mellado M., Jara C., Montenegro I., González S., Madrid A. (2015). Free radical-scavenging activity of sequential leaf extracts of Embothrium coccineum. Open Life Sci..

[90] Dudonné S., Vitrac X., Coutière P., Woillez M., Mérillon J.M. (2009). Comparative study of antioxidant properties and total phenolic content of 30 plant extracts of industrial interest using DPPH, ABTS, FRAP, SOD, and ORAC assays. J. Agric. Food Chem..

[91] Romay C., Pascual C., Lissi E.A. (1996). The reaction between ABTS radical cation and antioxidants and its use to evaluate the antioxidant status of serum samples. Braz. J. Med. Biol. Res..

[92] Alarcón E., Campos A.M., Edwards A.M., Lissi E., López-Alarcón C. (2008). Antioxidant capacity of herbal infusions and tea extracts: A comparison of ORAC-fluorescein and ORAC-pyrogallol red methodologies. Food Chem..

[93] Lopez-Alarcon C., Lissi E. (2006). A novel and simple ORAC methodology based on the interaction of Pyrogallol red with peroxyl radicals. Free Radic. Res..

[94] Griffith O.W. (1980). Determination of glutathione disulphide using glutathione reductase and 2-vinylpyridine. Anal. Biochem..

[95] Lutts S., Lefevre I., Delperee C., Kivits S., Dechamps C., Robledo A., Correal E. (2004). Heavy metal accumulation by the halophyte species Mediterranean saltbush. J. Environ. Qual..

[96] Vichai V., Kirtikara K. (2006). Sulforhodamine B colorimetric assay for cytotoxicity screening. Nat. Protoc..

[97] Skehan P., Storeng R., Scudiero D., Monks A., McMahon J., Vistica D., Warren J.T., Bokesch H., Kenney S., Boyd M.R. (1990). New colorimetric cytotoxicity assay for anticancer-drug screening. J. Natl. Cancer Inst..

